# Noses on the wing: the olfactory capacity of hawkmoth wings

**DOI:** 10.1242/jeb.252047

**Published:** 2026-07-28

**Authors:** Ahmed Reda Ismaieel, Regina Stieber, Bill S. Hansson, Sonja Bisch-Knaden

**Affiliations:** ^1^ Department of Evolutionary Neuroethology, Max Planck Institute for Chemical Ecology, Hans-Knoell-Straße 8, 07745 Jena, Germany; ^2^ Entomology Department, Faculty of Science, Ain Shams University, Abbassia, Cairo 11566, Egypt

**Keywords:** Olfaction, Sphingidae, Chemosensory sensilla, Ionotropic receptor, Wings, Amines

## Abstract

In addition to their primary olfactory organs, the antennae, insects can smell using other parts of their bodies. In this study, we examined the olfactory capabilities of the wings of the tobacco hawkmoth, *Manduca sexta* (Lepidoptera: Sphingidae). Using scanning electron microscopy, we identified an average of 21–32 sensory bristles along the margins of each wing. In addition to raised sockets that indicate mechanosensory function, we observed a subapical pore and numerous wall pores. These pores are signs of chemosensory function, suggesting that the wings can detect chemicals upon contact or in gaseous form. Gene expression analysis revealed the presence of genes encoding chemosensory receptors, such as gustatory receptors (GRs) and ionotropic receptors (IRs), including IR76b, the co-receptor of amine-sensing IRs, and IR8a, the co-receptor of acid-sensing IRs. Genes encoding several odorant receptors (ORs) were also expressed in the wings; however, expression of the gene encoding ORCo, the obligatory OR co-receptor, was not detected. Electrophysiological recordings revealed that only the volatile amines pyrrolidine and piperidine elicited responses from the wings. Protein modeling and molecular docking simulations identified members of the Lepidoptera-specific *IR7d* clade as encoding candidate receptors for these compounds. These are characteristic alkaloids found in solanaceous plants, which tobacco hawkmoths prefer for laying eggs. Together, these findings demonstrate that the wings of *M. sexta* function as accessory olfactory organs that may influence oviposition choice.

## Teaser

**Highlighted Article:** Tobacco hawkmoths use their wings to smell the alkaloids of their favorite host plants.

## INTRODUCTION

Insect wings carry numerous sensory sensilla, which are primarily located on the wings' base, margins and veins. Some of these sensilla act as proprioceptors and are dome shaped, while others detect airflow and vibrations, and have a hair-like structure (Dickinson et al., 1997; Ai et al., 2010). These sensilla are essential for flight control because of their mechanosensory function. However, the hair-like sensilla on insect wings can also function as contact chemoreceptors. The chemosensory sensilla, or taste bristles, on the wing margin of the vinegar fly *Drosophila melanogaster* are characterized by a terminal pore and house four contact chemosensory neurons and one mechanosensory neuron (Stocker, 1994; Palka et al., 1979). These taste bristles respond to sweet and bitter molecules (Raad et al., 2016) and play a role in mating behavior by detecting long-chain cuticular hydrocarbons upon contact (He et al., 2019).

The two main families of chemosensory receptor genes involved in contact chemosensation are those encoding gustatory receptors (GRs) and ionotropic receptors (IRs). Members of both families have been shown to be expressed in the wings of various insects, including beetles (Pang et al., 2018), flies (He et al., 2019), aphids (Agnel et al., 2017) and moths (Ma et al., 2016). However, some GRs and IRs can also mediate olfaction (Silbering et al., 2011; Kumar et al., 2020; MacWilliam et al., 2018). Additionally, genes encoding odorant receptors (ORs), which detect volatile cues, have been reported to be expressed in insect wings (He et al., 2019; Pang et al., 2018; Ma et al., 2016; Agnel et al., 2017; Chen et al., 2022). Based on these expression data and a study showing that the wings of the yellow fever mosquito, *Aedes aegypti*, respond to gaseous chemicals (Yang et al., 2021), it appears that the insect wing is an olfactory organ in addition to its mechanosensory and gustatory functions. To test this hypothesis, we examined the tobacco hawkmoth, *Manduca sexta*, which was recently shown to express several *OR* genes, *IR* genes and *GR* genes in its wings (Tom et al., 2022). Apart from proprioceptive sensilla (Stanchak et al., 2024), however, sensilla on the wings of *M. sexta* have not yet been investigated.

Butterflies and moths have two types of hair-like sensilla on their wings: sensory scales (sensilla squamiformia) and sensory bristles (edge sensilla) (Hallberg et al., 1999). The slender sensory scales have longitudinal ridges on their pore-less surfaces and are seated in raised sockets. Sensory bristles are shorter and stouter than sensory scales, lack cuticular ridges and are also set in raised sockets (Yoshida et al., 2001; Yoshida and Emoto, 2010; Vogel, 1911; Clever, 1958). In some moth species, a subapical pore was observed next to the tip of the sensory bristles (Vogel, 1911). As sensilla with a single pore are believed to be involved in contact chemosensation, sensory bristles on the wings of Lepidoptera may serve a gustatory function in addition to a mechanosensory one, similar to taste bristles on the wings of Diptera (Stocker, 1994). An additional olfactory function of wing sensilla would require porous sensillum walls, a feature that has not been previously described. However, most studies on wing sensilla in Lepidoptera have focused on mechanosensation (Yoshida, 2025), so the presence of wall pores may have been overlooked.

In this study, we examined the morphology, electrophysiological properties and chemosensory gene expression of the wings of *M. sexta* to explore their olfactory capacity.

## MATERIALS AND METHODS

### Insect rearing

*Manduca sexta* (Linnaeus 1763) caterpillars were reared on an artificial diet (Grosse-Wilde et al., 2011) and maintained at 26°C during the 14 h light phase and 24°C during the 10 h dark phase, with relative humidity at 60%. Male and female pupae were transferred to separate climate chambers (25°C), with relative humidity at 60% during the 16 h light phase and 70% during the 8 h dark phase. The emerged adults were collected daily and kept individually in brown paper bags (17 cm×26 cm) within the pupal chambers.

### Scanning electron microscopy

We detached the forewings and hindwings at their bases, and removed the scales by wiping the wings with a wet tissue. Then, we cut the wing margins using scissors. Samples were dehydrated by repeatedly washing them in 70% ethanol and placing them on tissue to air dry. Next, we transferred the samples to a second tissue and folded it over to protect the wing margins from damage. Finally, we mounted the wing margins on a holder covered with adhesive tape, sputter-coated them with gold, and examined them with a scanning electron microscope (LEO 1450 VP, Zeiss, Jena, Germany).

### Tissue collection and RNA extraction

We investigated the expression of chemosensory receptor genes in the wing margins of 3 day old virgin male and female *M. sexta*. To extract the RNA, we pooled the forewing and hindwing margins of a single moth to create one sample. We prepared three biological replicates for each group (male and female). The wings were excised and ground into a fine powder using a mortar and 1.5 ml TRI Reagent (Sigma-Aldrich) in liquid nitrogen. The resulting homogenate was transferred to a 2 ml Eppendorf tube. Subsequent steps followed the manufacturer's instructions for the Direct-zol RNA Miniprep Kit. The total RNA concentration obtained per sample ranged from 30 to 40 ng μl^−1^.

### NanoString gene expression assay

We used the nCounter XT CodeSet gene expression assay (NanoString Technologies, Inc., Bothell, WA, USA) to study chemosensory gene expression. The custom CodeSet (Zhang et al., 2022) included 268 probes targeting the genes encoding 71 ORs, 29 IRs, 49 GRs, 47 odorant-binding proteins, 5 pickpocket proteins and 3 sensory neuron membrane proteins, and 62 candidate reference gene transcripts. The experimental procedures and analyses have been described previously (Ismaieel et al., 2025; Tom et al., 2022). We used *Msex2_00104RA*, *Msex2_12610RA* and *Msex2_13433RA* as endogenous reference genes.

### Reverse-transcription PCR

To clarify the expression of the genes encoding the OR co-receptor ORCo and the IR co-receptors IR8a, IR25a and IR76b in the wings, RNA extracted from female wings (wing margins and remaining wings separately) and from female antennae (positive control) was used to synthesize cDNA with the Superscript III Reverse Transcriptase Kit (Thermo Fisher Scientific). To amplify the genes, PCR was performed with Phusion™ High-Fidelity DNA Polymerase (New England Biolabs) according to the manufacturer's protocol and the primers shown in Table 1 at an annealing temperature of 60°C. The size of the PCR products was visualized and analyzed with gel electrophoresis.

**Table 1.** PCR primers for gene amplification

Gene	Forward primer	Reverse primer
*MsexORCo*	ATGATGGCCAAAGTGAAAACACAGG	CTATTTCAGCTGCACCAACACCATG
*MsexIR8a*	AAGAGCAGTGAAAGAGAAGTTAGTGCGC	TCCACACCCTGTAAAGTGTGTCTTCTG
*MsexIR25a*	ATGTTATCAGCGAAAAAGACTCCTCACGTC	TCAAAATTTAGGTTTCAAATTAGATAAACCTAAATTTC
*MsexIR76b*	ATGGCCGGGATCGAGCTCATTATATC	TTATCGATACAGAAAAGCAGAAGGCGCTC

### Electrowingography

Electrowingography (EWG) (a modification of electroantennography, EAG) was used to measure olfactory responses from the wings. An excised hindwing was trimmed to fit between two recording electrodes, and attached to the electrodes with conductive gel (Spectra 360 electrode gel, Parker Laboratories). A continuous flow of charcoal-filtered, humidified air (0.5 l min^−1^) was directed through an 11 cm long aluminium tube with its outlet positioned 1–2 cm from the wing's dorsal surface. For odor stimulation, 10 μl of the test odorant was applied to a filter paper disk (1.2 cm in diameter) placed inside a glass Pasteur pipette. The pipette's tip was inserted into a small opening in the aluminium tube to deliver a 200 ms pulse of odor-enriched air (0.4 l min^−1^) into the main airstream. EWG signals were digitally converted using an IDAC-4 USB system (Syntech, Buchenbach, Germany) and processed using Autospike (Syntech).

To analyze the responses, we measured the maximum deviation from the baseline (i.e. the amplitude of the response). Then, we subtracted the average amplitude elicited by two control stimulations (performed at the beginning and end of each sequence of odor stimuli) from the amplitude elicited by each odor stimulus. This provided the solvent-subtracted EWG response.

### Odor stimuli

Odorants were diluted in 1:100 in hexane: pyrrolidine (CAS number 123-75-1), piperidine (110-89-4), pyrrole (109-97-7), pyridine (110-86-1), 2-acetylpyridine (1122-62-9), nicotine (54-11-5), trimethylamine (75-50-3), putrescine (110-60-1), spermidine (124-20-9), (±)-linalool (78-70-6), methyl hexanoate (106-70-7), isovaleric acid (503-74-2) and acetic acid (64-19-7).

Headspace was collected from a non-flowering *Datura wrightii* plant, a single *D. wrightii* flower and a flowering *Nicotiana attenuata* plant. Each plant or flower was placed in a polyethylene terephthalate bag (Toppits, Minden, Germany). Charcoal-filtered air was pumped into the bag through a silicone tube connected to a custom-made pump. The odor-enriched air exited the bag through a second silicone tube that passed through a volatile collection trap (Porapak-Q 25 mg, https://www.volatilecollectiontrap.com). Collection of volatiles was done in a climate chamber at 25°C (day) and 22°C (night), and relative humidity at 57% during the 14 h light phase and 65% during the 10 h dark phase. After 24 h, the traps were removed and eluted with 400 μl hexane.

### Molecular docking

The amino acid sequences of the candidate ORs and IRs retrieved from UniProt (https://www.uniprot.org/) were used to generate 3D models with the AlphaFold Protein Structure Database (https://alphafold.ebi.ac.uk). The structural models were selected if predicted Local Distance Difference Tests (pLDDT) yielded scores ≥80%. The predicted 3D structures were downloaded in PDB format and converted to PDBQT format using AutoDockTools v1.5.7. Ligand structures were downloaded from the PubChem database in 3D format and also converted to PDBQT format using AutoDockTools v1.5.7. Docking simulations were carried out using AutoDock, which applies the Lamarckian Genetic Algorithm to explore binding conformations. The receptor structures were treated as rigid, while the ligand torsions were given full flexibility (Morris et al., 2008). We defined the putative ligand-binding pocket by aligning the modeled receptor structures with the crystal structures of the jumping bristletail odorant receptor MhOR5 (PDB ID: 7LIG) for ORs and the rat ionotropic glutamate receptor iGluR2 (PDB ID: 1FTJ) for IRs. The docking grid was centered on this pocket to ensure physiologically relevant binding predictions. After docking, the top-ranked binding poses were analyzed further.

### Statistics and figure preparation

Sample size and statistical tests are described in both the text and figure legends. The significance level alpha was set to 0.05. Statistical analyses were done with GraphPad InStat (v3.10, GraphPad Software, San Diego, CA, USA). Figures were generated using PAST (v3.26; Hammer et al., 2001) and RStudio, and edited using Adobe Illustrator CS5.

## RESULTS

### Chemosensory sensilla on the wing margins of *M. sexta*

**Fig. 1.**
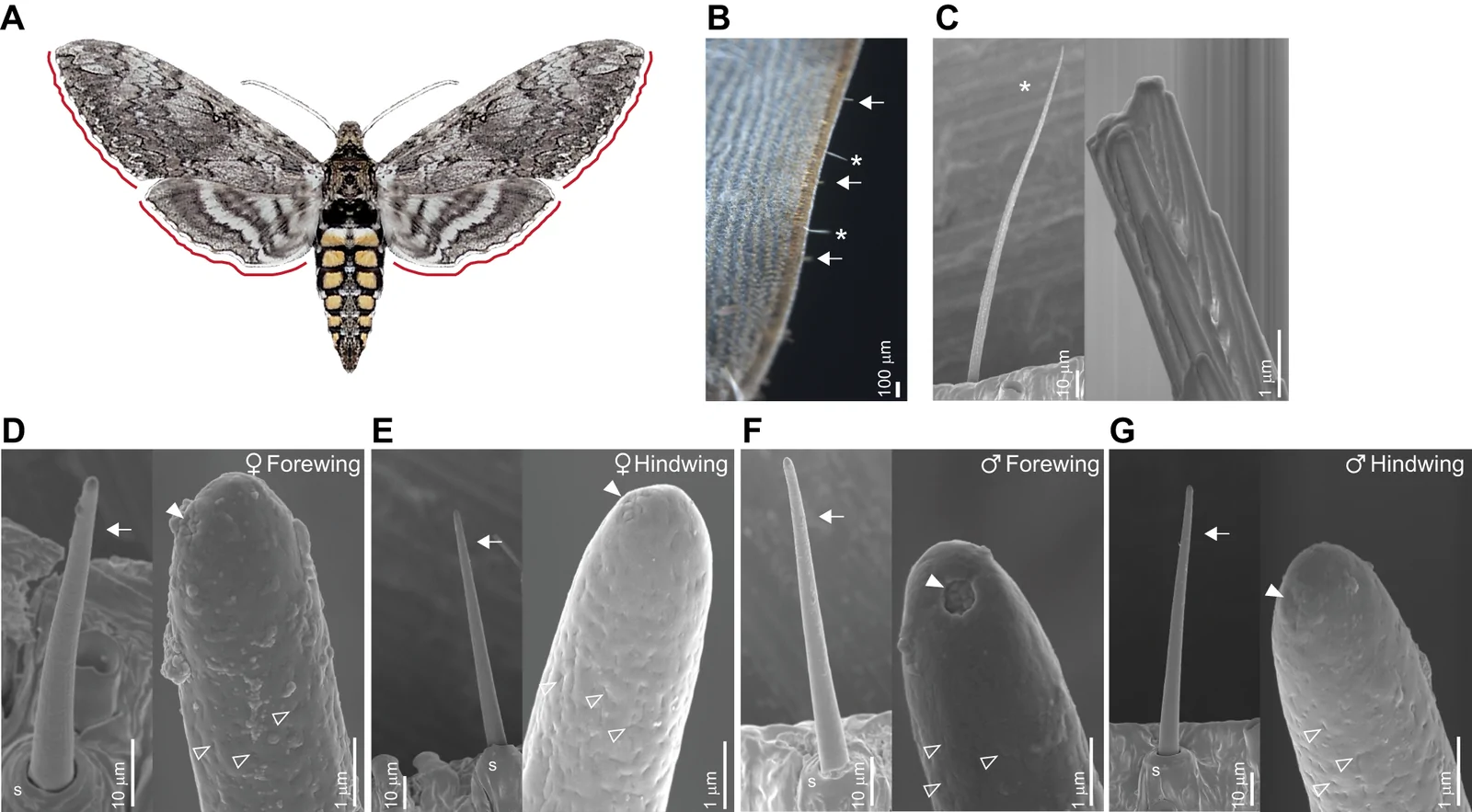
**Sensilla on the wing margins of *Manduca sexta*.** (A) A female *M. sexta*. Red lines mark regions along the wing margins where sensory scales and sensory bristles were found. (B) Light microscopy image of the margin of a female forewing. Arrows, sensory bristle. Asterisks, sensory scale. (C) Left: scanning electron microscopy (SEM) image of a sensory scale (asterisk). Right: enlarged view of the tip. (D–G) SEM images of sensory bristles on the forewing and hindwing margins of a female (D,E) and a male (F,G). Left: sensory bristles (arrows). S, socket. Right: enlarged view of bristle tips (filled arrowheads, subapical pores; open arrowheads, pores).

We used light and scanning electron microscopy to distinguish the two types of hair-like sensilla typically present on the wings of Lepidoptera (sensory scales and sensory bristles). These sensilla occurred alternately along the lateral margins of the forewings and the lateral and posterior margins of the hindwings of *M. sexta* (Fig. 1A,B). The wing margins (Fig. 1A, red lines) were longer in females than in males (forewings: 27±2 mm versus 23±2 mm, hindwings: 31±1 mm versus 26±2 mm, means±s.d.; *P*<0.001, *t*-test, *n*=8). One sensillum type was approximately 120 μm long with longitudinal ridges on its surface and a pointed tip (Fig. 1C). This type of sensillum occurred in similar numbers (on average, 10 to 17) on the margins of the forewings and hindwings of females and males (Table 2), and appears to represent sensory scales. The second type averaged 80 μm in length, ranging from 40 to 110 μm. It had a pitted surface and a pore located laterally below its blunt tip (Fig. 1D–G). This type of sensillum is likely to be the sensory bristles earlier described on the wings of Lepidoptera. An average of 21–32 of these sensilla were found on the margins of the forewings and hindwings, with a higher number on the hindwings of females than on those of males (Table 2). The subapical pore of these sensory bristles is characteristic of gustatory sensilla, while the basal socket (Fig. 1D–G) is characteristic of mechanosensory sensilla. The porous cuticle of the sensillum wall suggests an additional olfactory function.

**Table 2.** Sensilla on the wing margins of *Manduca sexta*

Sensillum type	Position	Females	Males	*P*-value
Sensory scale	Forewings	17±5	14±3	0.29
	Hindwings	11±2	10±2	0.21
Sensory bristle	Forewings	32±5	27±5	0.21
	Hindwings	28±2	21±5	0.02

Data are means±s.d. (*n*=5 for both males and females). *P*-values are from two-sided *t*-tests.

### Expression of chemosensory receptors in the wings

**Fig. 2.**
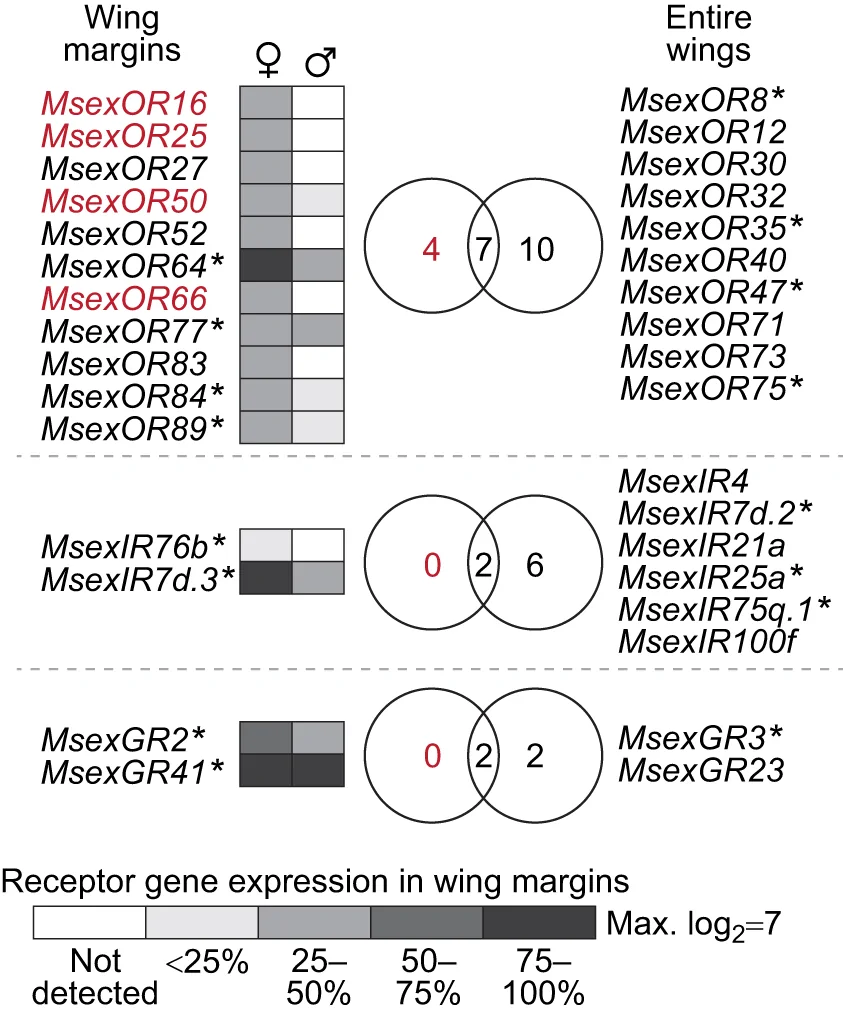
**Expression of chemosensory receptor genes in the wings of *M. sexta*.** Grayscale heatmaps show the expression of genes encoding odorant receptors (ORs), ionotropic receptors (IRs) and gustatory receptors (GRs) in the wing margins of virgin females and males. Margins of both forewings and hindwings were pooled for each animal. Cells illustrate the normalized log_2_ of the geometric mean (*n*=3 biological replicates) of normalized counts obtained from the NanoString assay (see Dataset 1). Receptors are shown in the heatmap if they are considered to be expressed in at least one sex (i.e. counts above background level in at least 2 of the 3 biological replicates). Light to dark shades of gray indicate low to high expression levels; white cells indicate no detection of transcripts (i.e. counts at background level in all 3 biological replicates). Asterisks indicate the receptor gene transcript was detected in at least eight out of nine previously tested appendages (Tom et al., 2022). Red indicates receptor genes expressed in wing margins, but not in entire wings. Venn diagrams show the total number of *OR*, *IR* and *GR* genes expressed in wing margins (see heatmaps) and entire wings (data for virgin females and males from Tom et al., 2022), and the number of receptor genes that are shared or unique. Receptor genes expressed in the entire wings, but not in the margins, are listed to the right of the Venn diagrams.

We found sensory bristles with a potential chemosensory function along the wing margins and expected chemosensory receptors to be expressed specifically in this region. Thus, we measured the expression levels of receptors belonging to the three chemosensory gene families (*OR*, *IR* and *GR* genes) in the wing margins of female and male moths using NanoString technology. We then compared these data with previous expression results obtained from entire wings (Fig. 2). Overall, we detected the expression of 15 receptor genes in the wing margins, which is half the number detected in entire wings (Tom et al., 2022). As with the entire wings, none of the receptors expressed in the margins exhibited sex-biased expression (*P*>0.5, DESeq2).

Eleven of the receptors expressed in the wing margins are encoded by the *OR* gene family. Four of these *OR* genes were detected only in the wing margins, not the entire wing, while 10 *OR* genes were previously found only in entire wing tissue. However, *ORCo* expression, which is required for functional ORs in higher insects, was not detected in the wings. This absence of *ORCo* expression in the wings was confirmed by RT-PCR (Fig. S1).

Unlike ORs, no receptors specific to the wing margins were found among the *IR* and *GR* gene families. Two *IR* genes were expressed both in the wing margins and the entire wing. One gene encodes the IR co-receptor IR76b, which mediates olfactory responses to amines in *D. melanogaster* (Vulpe and Menuz, 2021), whereas the other gene belongs to a Lepidoptera-specific *IR* gene clade (van Schooten et al., 2016) and encodes MsexIR7d.3. Six additional *IR* genes were expressed in the entire wing. Among these was the gene encoding the ubiquitous IR co-receptor IR25a, which, like IR76b, is involved in detecting amines. IR8a, the IR co-receptor associated with detecting acids, was not expressed in wing tissues according to the NanoString assay, but it was detected using the more sensitive RT-PCR (Fig. S1).

Additionally, two *GR* genes, *MsexGR2* and *MsexGR41*, were expressed in the wing margins and the entire wing. Two additional *GR* genes, *MsexGR3* and *MsexGR23*, were expressed in the entire wing tissue. *MsexGR41* had the highest expression level among the wing margin receptors and belongs to the bitter receptors (Koenig et al., 2015). Taken together, the expression patterns of chemosensory receptor genes in *M. sexta* wings suggest that this tissue can detect bitter substances upon contact, and volatile amines and potentially acids through olfaction.

### Electrophysiological recordings from the wings

**Fig. 3.**
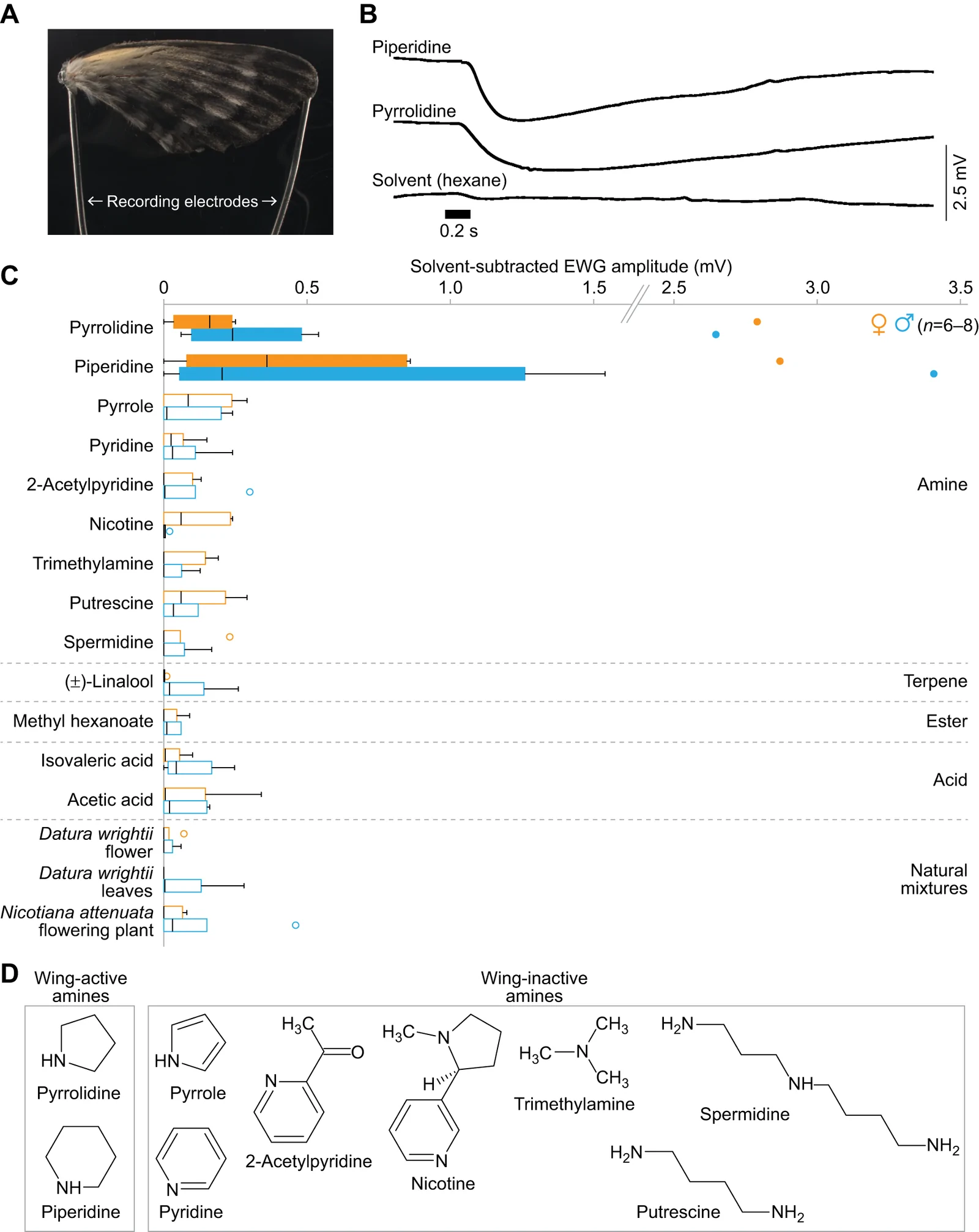
**Electrowingography (EWG).** (A) Photograph of a *M. sexta* hindwing attached to recording electrodes with conductive gel. (B) Representative EWG recordings of a male hindwing, with a 0.2 s odorant stimulus (piperidine, pyrrolidine or solvent control). (C) Solvent-subtracted EWG responses of female (orange) and male (blue) *M. sexta* hindwings to 13 synthetic odorants (10 μl, diluted 1:100 in hexane), and headspace of three natural odor sources (10 μl) (see Dataset 1). Boxplots show median, interquartile range and range; circles are outliers. Filled boxes indicate data significantly different from zero (*P*<0.05, Wilcoxon signed rank test, two-sided). (D) Molecular structures of wing-active and wing-inactive amines.

As we were interested in potential olfactory capabilities of the wings of *M. sexta*, we recorded odor-evoked responses using a technique similar to electroantennography (EAG), called electrowingography (EWG) (Yang et al., 2021). We recorded from the hindwings because they are smaller and easier to handle than the forewings (Fig. 3A). As the genes encoding the IR co-receptors IR76b and IR25a were found to be expressed in the wings (Fig. 2), indicating their ability to detect amines, we tested nine structurally diverse amines. The presence of *Ir8a* transcripts (Fig. S1) also indicates a sensitivity to acids. The absence of *ORCo* expression in the wings suggests that they might not be able to sense other odorants, such as terpenes, esters and aromatics. Nevertheless, we performed EWG recordings using representative odorants from these chemical classes, as well as natural blends from *M. sexta*’s nectar sources and host plants.

We found that the hindwings of male and female moths only showed an odor-specific response to pyrrolidine and piperidine (Fig. 3B). These responses were concentration dependent with a threshold at a dilution of 1:1000 (Fig. S2; see also Dataset 1). Structurally similar amines, such as pyrrole and pyridine, as well as the other stimuli tested, did not elicit a response (Fig. 3C,D). As the entire hindwing was used in these experiments, we could not determine whether the margin of the wing, where wall-pore sensilla were found (Fig. 1), was necessary for the observed response. Therefore, we performed additional EWG recordings using hindwings from which we had cut off the margins. Interestingly, the surgery did not affect the response to pyrrolidine and piperidine (entire versus marginless hindwing, *P*>0.4 for females and *P*>0.6 for males, Mann–Whitney-*U*-test; Fig. S3; see also Dataset 1). These results suggest that sensory bristles tuned to detect volatile saturated cyclic amines are present beyond the wing margins.

### Candidate amine-sensing receptors

Our gene expression analyses (Fig. 2; Fig. S1) and previous work (Tom et al., 2022) did not detect *MsexORCo* expression in the wings. However, several *OR* genes are expressed in this tissue and pyrrolidine activates two *D. melanogaster* ORs (Hallem et al., 2004). Therefore, we tested the hypothesis that the observed electrophysiological response to amines is mediated by ORs. Nevertheless, as amines are primarily detected by IRs (Silbering et al., 2011), we also included the IRs that, along with the co-receptors IR25a and IR76b, are expressed in *M. sexta* wing tissues (Fig. 2). Using AlphaFold2, we first predicted the structures of these proteins and estimated the reliability of the predictions using the pLDDT score, which ranges from 0 to 100, with higher values indicating a better prediction. We found that six MsexORs and four MsexIRs had pLDDT scores of at least 80.

We then performed molecular docking simulations to predict how these ten receptor proteins would interact with pyrrolidine and piperidine. As these two ligands are both strong bases, we expected the simulations to reveal hydrogen bonds with the acidic amino acid residues Asp (aspartic acid) and/or Glu (glutamic acid). Because the two active amines are closely related in structure, we also expected them to interact similarly with a given receptor.

**Fig. 4.**
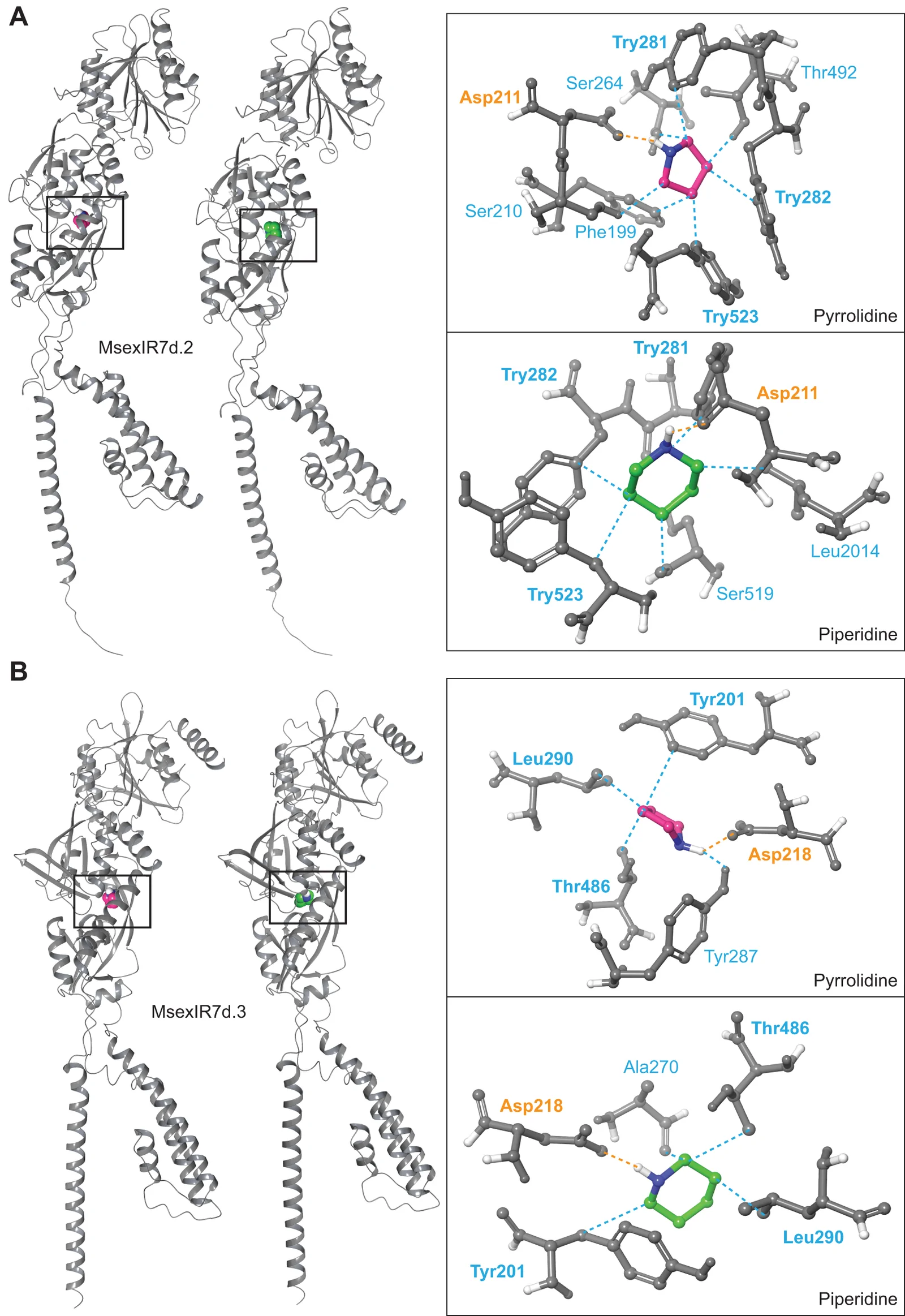
**Molecular docking simulations with candidate amine-sensing MsexIRs.** Left: predicted structures of MsexIR7d.2 (A) and MsexIR7d.3 (B) in pyrrolidine-bound (left) and piperidine-bound (right) states. Boxes indicate the position of the binding pocket. Right: view of the binding pocket with the top poses for pyrrolidine (top) and piperidine (bottom). Dashed lines indicate hydrogen bonds (orange) or other interactions (blue). Bold residues are conserved top poses for the two ligands.

The predictions for binding the two ligands to the MsexORs and to MsexIR75q.1 and MsexIR100f were weak and varied, suggesting non-specific or transient binding (Table 3). However, within the binding pockets of MsexIR7d.2 and MsexIR7d.3, the same acidic residues (Asp211 and Asp218, respectively) were predicted to form conserved hydrogen bonds with both pyrrolidine and piperidine. Furthermore, MsexIR7d.2 and MsexIR7d.3 exhibited three overlapping predicted interactions with the two chemically similar ligands (Fig. 4, Table 3). These results suggest that MsexIR7d.2 and MsexIR7d.3 could function as pyrrolidine- and piperidine-responsive receptors in the wings of *M. sexta*.

**Table 3.** Predicted interactions between wing-expressed receptors and wing-active amines

Receptor	Ligand	Hydrogen bonds	Other interactions
MsexOR30	Pyrrolidine	–	Thr52
	Piperidine	–	Val154, Gln172, Ala199
MsexOR47	Pyrrolidine	–	Val198, Val199, Leu301
	Piperidine	Glu192, Asn297	Phe298
MsexOR50	Pyrrolidine	–	Phe40
	Piperidine	Ser74, Thr78	Ile45, Val182
MsexOR64	Pyrrolidine	–	–
	Piperidine	Ser74	Val170
MsexOR83	Pyrrolidine	–	Tyr121, Phe8
	Piperidine	–	Phe46, Tyr76, Tyr166, Leu197
MsexOR84	Pyrrolidine	Gln9	Tyr26
	Piperidine	Tyr26, Asn215	Phe32, Leu222
MsexIR75q.1	Pyrrolidine	Glu566	Ile603
	Piperidine	Asp348	Ile329, Ser331, Trp351, Trp377
MsexIR100f	Pyrrolidine	Tyr257, Glu441	Arg291, Ile440, Lys290, Ser288, Val260
	Piperidine	–	Gly283
MsexIR7d.2	Pyrrolidine	Asp211	Tyr281, Tyr282, Tyr523, Phe199, Ser210, Ser264, Thr492
	Piperidine	Asp211	Tyr281, Tyr282, Tyr523, Leu214, Ser519
MsexIR7d.3	Pyrrolidine	Asp218	Leu290, Thr486, Tyr201, Tyr287
	Piperidine	Asp218	Leu290, Thr486, Tyr201, Ala270

## DISCUSSION

Through a combination of morphological, electrophysiological, gene expression and molecular docking experiments, we revealed that the wings of the hawkmoth *M. sexta* function as olfactory appendages. First, we confirmed the presence of sensory bristles on the lateral and posterior margins of the wings. These bristles are seated in raised sockets characteristic of mechanosensory sensilla and have been described along the wing margins of several butterfly and moth species (Yoshida, 2025; Clever, 1958; Vogel, 1911). Previous electrophysiological experiments have shown that sensory bristles on the wing margins of the silkmoth *Bombyx mori* are sensitive to vibrations and may therefore be involved in maintaining the moth's wingbeat (Ai et al., 2010).

We found that the sensory bristles along the margins of the wings of *M. sexta* have a subapical pore, suggesting a contact-chemosensory function (Hallberg et al., 1999). The mechanosensory and gustatory functions of sensory bristles on the moth wings are reminiscent of the bimodal taste bristles on Diptera wings (Stocker, 1994). However, we revealed that the sensory bristles of *M. sexta*, in addition, have porous surfaces. This morphological feature has not yet been described for sensilla on the wings of Lepidoptera. It is, however, characteristic of sensilla with an olfactory function (Hallberg et al., 1999). Thus, our data suggest that the sensory bristles of *M. sexta* may be trimodal. These wing bristles closely resemble the ‘scattered blunt sensilla’ found on the legs of hawkmoths, which have a similarly shaped subapical pore and porous surfaces (Kent and Griffin, 1990), supporting the idea that insect wings evolved from legs (Dickinson et al., 1997).

Electrophysiological recordings using volatiles from different chemical classes showed that the hindwings of *M. sexta* respond specifically to stimulation with two amines, pyrrolidine and piperidine. As sensory bristles have only been described at the margins of Lepidoptera wings (Hallberg et al., 1999; Vogel, 1911; Clever, 1958; Yoshida, 2025), we expected wings without margins to no longer respond to olfactory stimuli. However, wings with cut margins exhibited the same response as intact wings. This finding suggests that sensory bristles may be present on wing surfaces as well. However, despite thorough investigation, no sensory bristles or other potential olfactory sensilla were identified on the wing surfaces, which are densely covered with scales. The average length of these wing scales in hawkmoths is 170 μm (Simonsen and Kristensen, 2003), approximately twice the length of the sensory bristles on the wing margins of *M. sexta*. This size difference may explain why olfactory sensilla on the wing surface have not yet been detected. Notably, sensilla with an apical pore and a putative chemosensory function have been found on the wing surface of the migratory locust, which has no scales on its wings (Zhou et al., 2008). Another indication of the presence of sensory bristles on areas of the wings other than the margins is that the margins inevitably sustain damage during flight. For example, the painted lady butterfly, *Vanessa cardui*, experiences the most significant wing damage on the lateral margins of the forewings and the posterior margins of the hindwings (Korkmaz et al., 2023), i.e. the locations where sensory bristles have been documented in Lepidoptera. However, insects like the hummingbird hawkmoth, *Macroglossum stellatarum*, can compensate for wing damage by increasing their wingbeat amplitude and frequency (Kihlström et al., 2021), behaviors that are mediated by sensory bristles (Ai et al., 2010). Together, these data and our current findings suggest that sensory bristles and their ability to sense mechanosensory and olfactory cues are not limited to the margins, which are the most vulnerable part of the wings.

A comparison of the molecular structures of the tested amines that elicited and did not elicit a response revealed that only the saturated cyclic amines pyrrolidine and piperidine were wing active. In contrast, their unsaturated counterparts (pyrrole and pyridine) and odorants from other chemical classes were inactive. This response profile suggests that the responsible receptors are narrowly tuned to amines with distinct molecular properties. Responses to amines are mediated by chemosensory receptors that belong to the IR family. In *Drosophila*, IR25a and IR76b act as co-receptors for amine-sensing IRs (Vulpe and Menuz, 2021; Silbering et al., 2011). Genes for both co-receptors were expressed in the wings of *M. sexta*, consistent with the expression pattern of other moth species, including *Bombyx mori*, *Spodoptera litura* and *Helicoverpa armigera* (Liu et al., 2018; Yin et al., 2021; Zhu et al., 2018). The obligatory OR co-receptor gene *ORCo* was not detected in the wings of *M. sexta*, despite the expression of several *OR* genes. This finding aligns with expression data from the wings of the moth *Ectropis obliqua* (Ma et al., 2016). The wing-expressed *MsexOR* genes were not likely to encode receptors that could bind the active amines, as demonstrated by molecular docking simulations. They may therefore be involved in internal, ORCo-independent cellular signaling functions.

Of the six tuning *IR* genes expressed in the wings, two – *MsexIR7d.*2 and *MsexIR7d.3* – were identified as encoding potential pyrrolidine and piperidine receptors via protein modeling and molecular docking simulations. Both belong to the Lepidoptera-specific *IR7d* clade, which remains functionally uncharacterized (van Schooten et al., 2016; Yin et al., 2021). Molecular docking simulations help develop hypotheses about which receptors may detect specific ligands (Comte et al., 2025). However, expressing these *IR* genes in a heterologous expression system, such as *Xenopus* oocytes (Hou et al., 2022), is necessary for robust functional characterization. Previous studies have shown that members of the *MsexIR7d* clade are expressed in all of the tested body appendages, including the antennae, wings, mouthparts, legs and ovipositor (Klinner et al., 2016; Tom et al., 2022). Functional data from the epiphysis, a recently described olfactory organ located on the tibia of the foreleg (Ismaieel et al., 2025), and from the ovipositor (Klinner et al., 2016) revealed that pyrrolidine is a strong ligand, which is consistent with our current findings regarding the wings. Together, these physiological results and the ubiquitous expression pattern of candidate amine-sensing IRs suggest that the ability to detect amines with non-antennal body appendages is essential to the life history of *M. sexta*.

However, the impact of odor-evoked wing activation on behavior remains unknown. Sensory neurons from the wing base of hawkmoths target not only the thoracic ganglia but also the subesophageal ganglion in the head (Ando et al., 2011). This suggests that sensory neurons from more distal wing regions may have central projections to the subesophageal ganglion as well, where different sensory inputs may converge and influence behavior. Although pyrrolidine alone did not trigger feeding or oviposition behavior in female *M. sexta* in wind tunnel experiments (Bisch-Knaden et al., 2018), the odor may contribute to identifying suitable oviposition sites when part of a natural plant bouquet. *Manduca sexta* caterpillars are specialist feeders on solanaceous plants, including *Nicotiana*, *Datura* and *Solanum* species. These plants synthesize characteristic alkaloids, for which pyrrolidine and piperidine are important precursors (Cinelli and Jones, 2021; O'Hagan, 2000; Pomilio et al., 2008). Solanaceous plants contain high concentrations of alkaloids. However, these compounds were not found in the headspace of the leaves (Massadeh et al., 2022). Correspondingly, the wings of *M. sexta* did not respond to the leaf volatiles collected from undamaged plants. Nevertheless, pyrrolidine and piperidine may be released when a moth scratches the leaf surface with the spines on its tarsi while hovering in front of and inspecting the plant. Interestingly, the wings of *A. aegypti* respond most strongly to triethylamine among seven chemically diverse stimuli (Yang et al., 2021). This odor is an amine of plant origin and an oviposition attractant for mosquitoes (Leal et al., 2008). Our study reveals that the wings of the hawkmoth *M. sexta* are tuned to detect amine compounds present in the moth's preferred host plant family. Therefore, the wings may function as accessory olfactory organs involved in host plant-related behavior.

## Supplementary Material

10.1242/jexbio.252047_sup1 Supplementary information

Dataset 1. Raw data for Figs 2 and 3, and for Figs S2 and S3
