# Supplementary material for: Noses on the wing: the olfactory capacity of hawkmoth wings
Source: J Exp Biol. 2026 Jul 6;229(14):jeb252047. doi: 10.1242/jeb.252047 (PMC13380975; doi:10.1242/jeb.252047)
Supplement: Supplementary information [file jexbio-229-252047-s1.pdf]

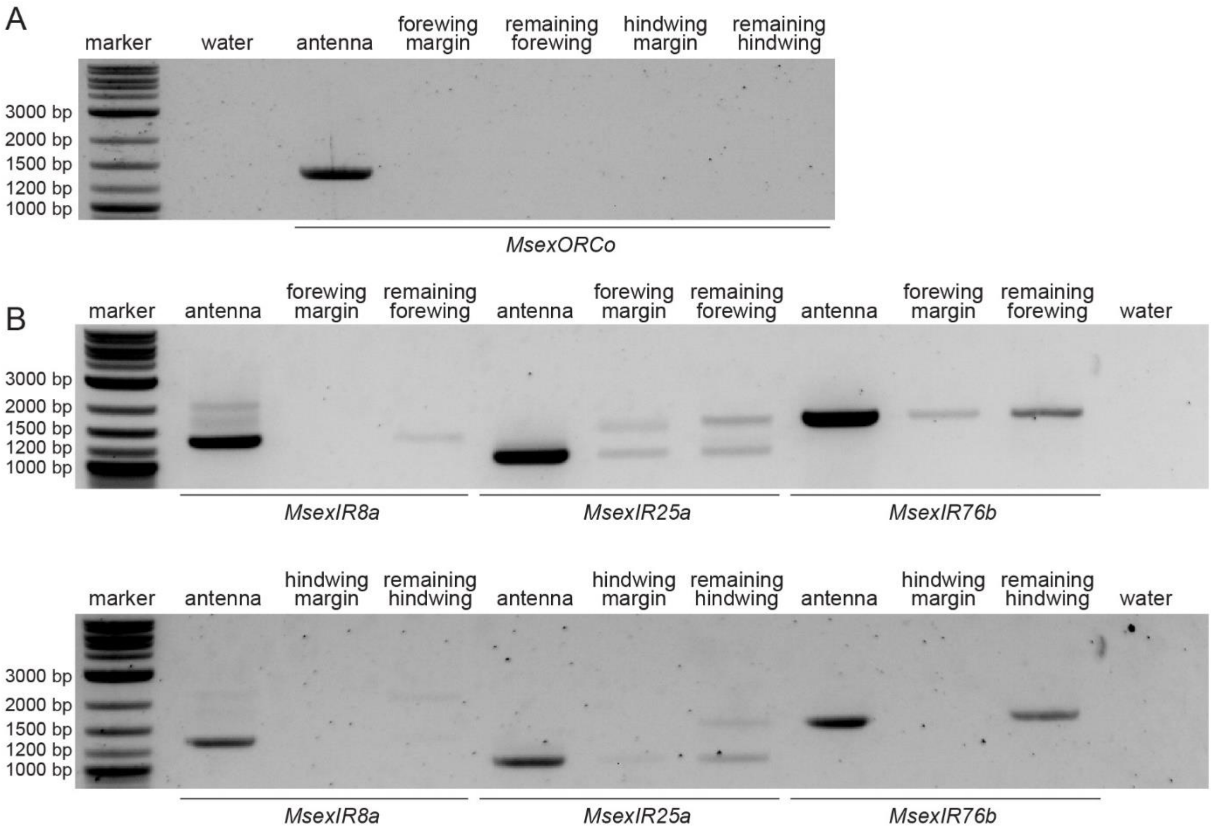

Fig. S1. Reverse-transcription PCR amplifications of the *OR* co-receptor *ORCo* (A) and the *IR*-coreceptors *IR8a*, *IR25a*, and *IR76b* (B) in the wings of females

RNA extracted from the antenna served as the positive control, and water as the negative control.

Journal of Experimental Biology • Supplementary information

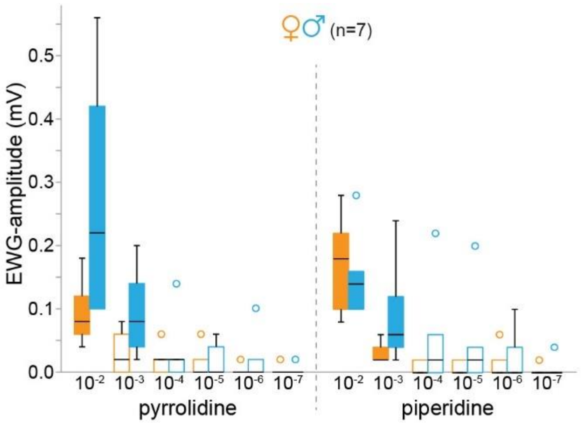

Fig. S2. Dose-Response EWG experiments

EWG responses of female (orange) and male (blue) *M. sexta* hindwings to pyrrolidine and piperidine (10 µl, diluted in hexane) (see Dataset 1 in the supplementary information) . *Boxplots*, median, interquartile range and range. *Circles*, outliers. *Filled boxes*, data different from zero ($p<0.05$, Wilcoxon signed rank test, two-sided).

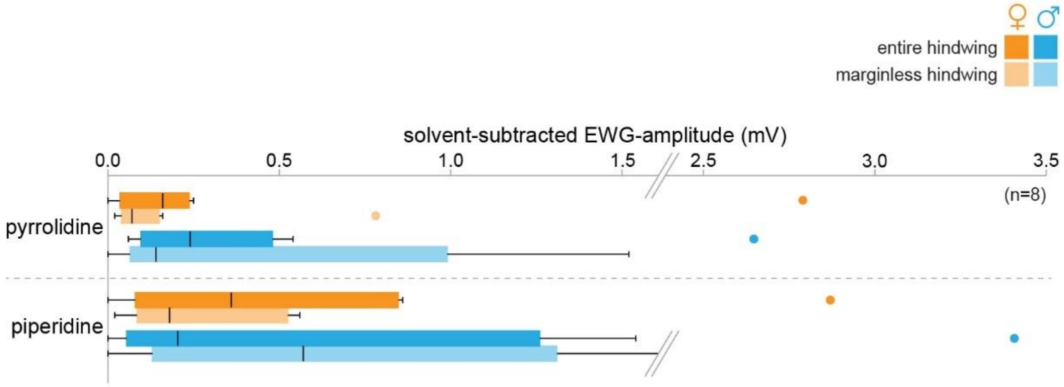

Fig. S3. EWG with and without wing margins

Solvent-subtracted EWG responses of female (orange) and male (blue) M. sexta hindwings to pyrrolidine and piperidine (10 µl, diluted 1:100 in hexane) (see Dataset 1 in the supplementary information). *Boxplots*, median, interquartile range and range. *Circles*, outliers. All data different from zero ($p<0.05$, Wilcoxon signed rank test, two-sided). Results from entire wings (solid color, same data as in Fig. 3C) were similar to results from marginless hindwings (faint color); $p>0.4$ for females and $p>0.6$ for males (Mann-Whitney U test, two-sided).

Dataset 1. Raw data for Figs 2 and 3, and for Figs S2 and S3

Available for download at
https://journals.biologists.com/jeb/article-lookup/doi/10.1242/jeb.252047#supplementary-data

Journal of Experimental Biology • Supplementary information
